# Dental Education in Germany

**Published:** 1888-01

**Authors:** W. D. Miller

**Affiliations:** Berlin


### DENTAL EDUCATION IN GERMANY.

BY W. D. MILLER, BERLIN.

In consideration of the recent awakening which has taken place on the subject of dental education and the praiseworthy efforts that are being made to raise the standard of dental requirements and the status of the dental profession, I readily acquiesce in the request of the editor of the INDEPENDENT PRACTITIONER to furnish some information on the subject of Dentistry and Dental Education in Germany. A comparison of the respective systems of education in different countries and the results accomplished by them must always be of interest and often of profit, particularly to those engaged in educational work, as well as to all those interested in the standing of the dental profession at home and abroad. It would not, however, be justifiable to say that the system of education which has produced the best results in Germany or England would likewise be best adapted to the need of the profession in America, or *vice versa*. The preparation of a dentist must, to a certain extent, be made to conform to the demands made upon him. I will return to this statement later on.

There are two very distinct classes of persons practicing dentistry in Germany: the Zahnärzte (tooth-doctors) and the Zahnkünstler (tooth-artists), or Zahntechniker (tooth-mechanics) or Zahnarbeiter (tooth-workmen), as some of the Zahnärzte insist upon calling them. The Zahnarzt occupies about the position of the dentist in America who has passed the dental examination imposed by a State board of examiners; I shall, however, use the term Zahnarzt untranslated, partly in order to avoid all confusion, and partly because every Zahnarzt is very proud of his title, and would feel deeply wronged to be styled dentist. I must confess that in this they cannot be blamed, as I myself do not allow the term dentist to appear in connection with my name, unless it is put there by some correspondent; not that I am ashamed of my profession, but that the title "American dentist" (or D. D. S.), through the unworthy action of some few of our regularly chartered dental colleges, has become one of such doubtful significance that I prefer not to have the accompanying suspicion attached to my name. I shall return to this question on another occasion.

The term Zahntechniker originally meant one who did mechanical work exclusively—mechanical dentist; now Zahntechniker, Zahnkünstler, etc., etc., are simply terms used by any one who chooses to employ them to signify that he is engaged in the practice of dentistry. Those who have not passed the State examination, and cannot therefore call themselves Zahnärzte, take any name which best suits their fancy, and practice what they please.

It is only since the year 1869 that the Zahntechniker have played a very prominent part in the history of German dentistry. Previous to that time, the practice was restricted by law to the Zahnärzte; that is, to certain persons who had fulfilled certain requirements, specified below, as to general and dental education, and had passed the examination in these subjects, imposed by the State.

In 1869 a law was enacted opening the practice of medicine and of dentistry to every one without reference to general or special education or fitness for the work. It naturally happened as a result of this condition of affairs, known as Gewerbe-Freiheit (literally, freedom of trade; in this case, freedom of practice), many turned their attention to dentistry who did not have the necessary preliminary education to admit them, either to the State dental examination, or to the study of dentistry at the University. These persons make up the class denominated Zahnkünstler, etc. In fact, any person who can master a few excavators and a vulcanizer may call himself Zahnkünstler, and begin work on the first one whom he can induce to trust to his skill.

From this it must not, however, be imagined that all the good work in Germany is done by the Zahnärzte and the bad by the Zahnkünstler. There are many of the latter who do very good work, and the better class of Zahnkünstler is, practically, in advance of the poorer class of Zahnärzte; and not infrequently the Zahnarzt yields to the Zahnkünstler in the struggle for existence. The Zahnkünstler, having been at much less expense of time and money than the Zahnarzt, and having begun practice earlier in life, also living usually at less expense, is able to do work at a lower (more reasonable ?) price than the Zahnarzt, and if he is skillful, as many of them are, he naturally takes the lead of the Zahnarzt, especially in practice,among the lower classes, if we may not also say the middle classes, where the first question is, what does it cost ? For this reason (as well as because this unlimited license of practicing dentistry has done a great deal to hinder the progress of this specialty) the Zahnarzt has for many years waged a bitter war against the Zahnkünstler, for the purpose of restricting him by law in the practice of dentistry.

Persons of German nationality, who purpose making the career of a Zahnarzt, are obliged, first to acquire the Reife für Prima of a German gymnasium, or first-class Real Schule; that is, they are obliged to pursue their studies at the gymnasium until they have passed the examination admitting them to Prima. This is called the Vorbildung, and is equivalent to that required to enter the Sophomore class at Ann Arbor or Yale. Secondly, they must furnish evidence of having studied four semesters at a German University, and thirdly, a certificate given by some Zahnarzt of having had practical exercise in mechanical dentistry. Fourthly, they must pass the State dental examination, as described below.

The practical American reader will, no doubt, at once say that one may have fulfilled all these conditions and still know very little about dentistry, and this is the case. The preliminary education (Vorbildung) is what it pretends to be. No one can work his way up to Prima without having applied himself diligently for many years in the acquirement of general knowledge, more particularly of Greek and Latin. The second condition, however, appears remarkably insufficient, nothing whatever being specified as to what is to be studied, it being immaterial whether it is law, theology, medicine or natural sciences, etc.,* or the student may spend the whole of the four semesters in the Kneipe, it being necessary for him to appear only once at the beginning and once at the end of the semester to secure the necessary certificate of attendance.

A certificate of having had practical exercise in mechanical dentistry is often, as may be easily supposed, obtained for very little work. It follows that candidates for the State dental examination in Germany must have had a good general education, but need know very little about dentistry. It has more than once happened that persons bearing the title "Praktischer Zahnarzt" have come to the Berlin institute to continue their studies who have never made a gold filling or adjusted the rubber-dam. Consequently,

---

At some Universities this law is now being so interpreted as to require attendance at medical or dental lectures for at least three of the semesters.

while the average German Zahnarzt possesses a much better general education than the average American dentist, in the matter of dentistry itself the latter is far ahead. Of course there are exceptions on both sides. I here speak of averages. I may also premise what I shall discuss more fully farther on, that the Zahnärzte at present turned out by the Dental Institute of the University of Berlin, while having a better general education, are, I think, nearly, I had almost said quite, as well prepared to practice dentistry in all its branches as the average American graduate.

The State dental examination may be made at any university in Germany. The candidate is examined in (1) Anatomy, (2) Physiology, (3 )Pathology, (4) General Surgery, (5) Special Surgery (of the head), (6) Materia Medica, (7) Toxicology, (8) Dentistry—operative, mechanical and scientific. These examinations are not always, however, all that they are supposed to be. At some universities they have been made notoriously easy, so much so that students at the Berlin Institute who, for any reason, generally lack of industry, are afraid to attempt the examination in Berlin, stay out the four semesters here and then go somewhere else to make their examinations. It consequently has often happened that candidates have passed the examinations knowing very little about medicine, and still less about dentistry. From this it will be seen that America is not the only country in which dental examinations may be easily made, and that there are Indian paths in Germany as well as in Philadelphia. Recent events, however, seem to indicate that the State dental examinations are being rendered more severe in other universities than Berlin.*

The manner in which the examinations are conducted I will describe in detail when I come to speak of the Dental Institute of the University of Berlin.

---

In America and Germany the systems of dental education have, until recently, been nearly diametrically opposed, the one requiring nothing but dentistry, more particularly practical dentistry, the other devoting many years to general education, and comparatively little time to dentistry itself. That both of these systems are extremes there is no question, and that of the two, the American, as best fitting the man for the performance of his duties, is the better, is equally indisputable. A dentist should be, above all other things, educated in dentistry, scientifically and practically, and should never sacrifice his dental to a general education. I am, however, by no means in favor of making dentists out of ignoramuses. What is most desirable is an amalgamation of the two systems, as we are trying to amalgamate them in Berlin, the general education required in Germany, followed by the practical education given in the best American colleges. This is, however, a broad question which I do not propose to discuss here.

As already stated, up to the year 1869, dentistry was practiced in Germany, with very few exceptions, by such persons only as had passed the examinations for and received the dental approbation. Some of the more successful Zahnärzte, however, employed as assistants in the mechanical laboratory persons who had not made the examination. These assistants received the name of "Techniker," and it is now claimed by the Zahntechniker that the reputation of many Zahnärzte was built up by the work of their Techniker alone.

In 1869, a law was enacted which opened the practice of medicine and dentistry to any and every one, entirely independent of ability or education. This condition of affairs (called the Gewerbe-Freiheit) made it possible for the Techniker to establish himself independently in practice, and they were not slow in taking advantage of the opportunity, as the present figures will show. In 1869 there were, in Berlin, some fifty Zahnärzte, and half a dozen Zahntechniker. In 1887 there are about sixty Zahnärzte, and not much under 200 Zahntechniker.

There appears, also, to be little doubt that the freedom to practice dentistry was and is employed to a very undesirable extent. Any one who had anywhere picked up the least smattering of dentistry, not only the regular Techniker from the mechanical laboratories, but servants, barbers, anybody could begin at any time to work on the human teeth. Not being allowed to call themselves Zahnärzte, they made use of all kinds of titles, such as Zahntechniker, Zahnkünstler, Zahnartist, Dentist, Dentiste, Lehrer der Zahnheilkunde, Zahnoperateur, Specialist für Zahnheilkunde, etc., etc., and proposed to perform all kinds of operations on the human teeth, not only the insertion of artificial teeth, but filling, extracting, etc., etc. Naturally the Zahnärzte were highly dissatisfied with this state of things, partly because of the havoc made in their practices by so many tooth-artists, and partly because the standing of the whole dental profession in Germany suffered, the public not being able to distinguish between the Zahnarzt and the Zahnartist. Thus originated the struggle between the Zahnärzte and the Zahnkünstler, which seems to gradually increase in intensity. The object of the Zahnärzte is to limit the Zahnkünstler in the practice of dentistry, or at least to compel them to drop the title Zahntechniker, unless they are able to fulfill certain conditions yet to be determined, while the Zahntechniker attribute the whole movement to *jalousie de métier* on the part of the Zahnärzte, and are putting forth all their strength to maintain their present rights, usually making a very good showing at exhibitions in mechanical and operative work, anatomical and microscopic preparations, etc., etc. They have also established night schools, and among their teachers have numbered no less a person than Robert Hartmann, professor of anatomy in Berlin. These educational attempts are considered by the Zahnärzte as only a blind to keep off adverse legislation.

I have been asked whether it is true that there are cities in Germany of 10,000 inhabitants with only one Zahnarzt. Yes, there are cities of 45,000 inhabitants with only one Zahnarzt; there are many places of 10,000 to 15,000 inhabitants with no Zahnarzt whatever, and I have been told of a city of 40,000 inhabitants, near Essen with no Zahnarzt and only one Zahntechniker. Here it is not at all unfrequent for people to travel two to ten hours by rail to go to their dentist. In those places having neither Zahnarzt nor Techniker, the extractions are performed by physicians, barbers, etc.

The organizations in Germany are pretty much the same as in America. There exists (1) a National Dental Society (Central Verein Deutscher Zahnärzte), meeting once a year at some place chosen at the previous meeting, and having active, honorary and corresponding members. Nearly every province has its local society; for example, Verein fur Rheinland und Westphalen, Verein Schleswig-Holsteiner Zahnärzte, etc., etc. Berlin has two local societies; the older one, Die Berliner Zahnärztliche Gesellschaft, and the younger one, Der Verein Deutscher Zahnärzte, the avowed object of the latter being to fight the Zahnkünstler and the American dentists. There is also, of recent origin, a society of German dentists holding only the degree D. D. S., called "Der Verein der in Amerika graduirten Doktoren der Zahnheilkunde." The following journals are published :

(1). Zahnärztliches Wochenblatt, - - Editor, Zahnarzt, Dr. Andreae.
(2). Deutsche Monatsschrift für Zahnheilkunde, " " Parreidt.
(3). Correspondenz Blatt, für Zahnärzte - " C. Ash & Sons.
(4). Journal für Zahnheilkunde, - - " E. Richter, D. D. S.
(5). Zahntechnische Reform - - - " Zahntechniker, Pawelz.
(6). Monatsschrift des Vereins Deutscher Zahnkünstler, Editor, Zahnkünstler Polscher.

(TO BE CONCLUDED.)

